# Mild to moderate post-COVID-19 alters markers of lymphocyte activation, exhaustion, and immunometabolic responses that can be partially associated by physical activity level— an observational sub-analysis fit- COVID study

**DOI:** 10.3389/fimmu.2023.1212745

**Published:** 2023-09-11

**Authors:** Bruna Spolador de Alencar Silva, Telmo Pereira, Luciele Guerra Minuzzi, Camila Souza Padilha, Caique Figueiredo, Tiago Olean-Oliveira, Ivete Vera Medeiros dos Santos, Ana Elisa von Ah Morano, Osmar Marchioto Júnior, José Procópio Jabur Ribeiro, Vanessa Ribeiro Dos Santos, Marília Seelaender, Alexandre Abílio Teixeira, Ronaldo Vagner T. Dos Santos, Valdir de Aquino Lemos, Ana Paula Coelho Figueira Freire, Gilson Pires Dorneles, Bruna Marmett, André Olean-Oliveira, Marcos F. S. Teixeira, Patrícia M. Seraphim, Armando Caseiro, Ricardo Aurino Pinho, Hashim Islam, Jonathan Peter Little, Karsten Krüger, José César Rosa-Neto, Manuel-João Coelho-E-Silva, Fábio Santos Lira

**Affiliations:** ^1^ Exercise and Immunometabolism Research Group, Postgraduation Program in Movement Sciences, Department of Physical Education, Universidade Estadual Paulista (UNESP), Presidente Prudente, Brazil; ^2^ Polytechnic Institute of Coimbra, Coimbra Health School, Coimbra, Portugal; ^3^ Postgraduation Program in Movement Sciences, Department of Physical Education, Universidade Estadual Paulista (UNESP), Presidente Prudente, Brazil; ^4^ Cancer Metabolism Research Group, LIM26-HC, FMUSP, Universidade de São Paulo, São Paulo, Brazil; ^5^ Department of Cell and Developmental Biology, University of São Paulo, São Paulo, Brazil; ^6^ Department of Biosciences, Universidade Federal de São Paulo (UNIFESP), Santos, Brazil; ^7^ Department of Health Sciences, Central Washington University, Ellensburg, WA, United States; ^8^ Physiotherapy Department, Universidade do Oeste Paulista (UNOESTE), Presidente Prudente, Brazil; ^9^ Laboratory of Cellular and Molecular Immunology, Graduate Program in Health Sciences, Federal University of Health Sciences of Porto Alegre, Porto Alegre, Brazil; ^10^ Department of Chemistry and Biochemistry, School of Science and Technology, Universidade Estadual Paulista (UNESP), Presidente Prudente, SP, Brazil; ^11^ Graduate Program in Health Sciences, School of Medicine, Pontifícia Universidade Católica do Parana, Curitiba, Brazil; ^12^ School of Health and Exercise Sciences, University of British Columbia, Kelowna, BC, Canada; ^13^ Department of Exercise Physiology and Sports Therapy, Institute of Sports Science, Justus-Liebig-University Giessen, Giessen, Germany; ^14^ Faculty of Sport Sciences and Physical Education, Research Center for Sport and Physical Activity (uid/dtp/04213/2020), Universidade de Coimbra, Coimbra, Portugal

**Keywords:** SARS-CoV-2, physical activity, immune response, metabolism, inflammation, post-acute COVID-19 syndrome

## Abstract

**Aim:**

This study aimed to evaluate if physical activity is associated with systemic and cellular immunometabolic responses, in young adults after mild-to-moderate COVID-19 infection.

**Methods:**

Mild- to- moderate post-COVID-19 patients (70.50 ± 43.10 days of diagnosis; age: 29.4 (21.9– 34.9) years; BMI: 25.5 ± 4.3 kg m^2^ n = 20) and healthy age-matched controls (age: 29.3 (21.2 – 32.6) years; BMI: 25.4 ± 4.7 kg m^2^; n = 20) were evaluated. Physical activity levels (PAL), body composition, dietary habits, muscular and pulmonary function, mental health, sleep quality, metabolic parameters, immune phenotypic characterization, stimulated whole blood and PBMC culture (cytokine production), mRNA, and mitochondrial respiration in PBMCs were evaluated.

**Results:**

The post-COVID-19 group exhibited lower levels of moderate to vigorous physical activity (MVPA) (p = 0.038); therefore, all study comparisons were performed with adjustment for MVPA. Post-COVID-19 impacted the pulmonary function (FEV1, FEV1%pred, FVC, and FVC %pred) compared with the control (p adjusted by MVPA (p adj) <0.05). Post-COVID-19 exhibited lower levels of serum IL-6 (p adj <0.01), whereas it showed higher serum IL-10, triglyceride, leptin, IgG, ACE activity, TNFRSF1A, and PGE_2_ (p adj <0.05) levels compared with controls. Post-COVID-19 presented a lower percentage of Treg cells (p adj = 0.03) and altered markers of lymphocyte activation and exhaustion (lower CD28 expression in CD8^+^ T cells (p adj = 0.014), whereas CD4^+^T cells showed higher PD1 expression (p adj = 0.037)) compared with the control group. Finally, post- COVID-19 presented an increased LPS-stimulated whole- blood IL-10 concentration (p adj <0.01). When exploring mitochondrial respiration and gene expression in PBMCs, we observed a higher LEAK state value (p adj <0.01), lower OXPHOS activity (complex I) (p adj = 0.04), and expression of the Rev-Erb-α clock mRNA after LPS stimulation in the post-COVID-19 patients than in the control (p adj <0.01). Mainly, PAL was associated with changes in IL-10, triglyceride, and leptin levels in the plasma of post-COVID-19 patients. PAL was also associated with modulation of the peripheral frequency of Treg cells and the expression of PD-1 in CD8+ T cells, although it abrogated the statistical effect in the analysis of TNF-α and IL-6 production by LPS- and PMA-stimulated PBMC of post-COVID-19 patients.

**Conclusion:**

Young adults after mild-to-moderate SARS-CoV-2 infection appeared to have lower physical activity levels, which can be associated with clinical and immunometabolic responses in a complex manner.

## Introduction

Despite the very low general mortality rate from COVID-19 in young/middle- aged individuals, there is a significant portion of the population recovered from COVID-19 that might develop multiple long-term health consequences (1). Persistent chronic symptoms are considerably present in post- SARS-CoV-2 infection and can be named as post-acute sequelae of SARS-CoV-2 infection (PASC) (commonly 3 months from the acute COVID-19, for a minimum of 2 months and cannot be related to another diagnosis) (2, 3). Frequent symptoms include fatigue, dyspnea, cognitive and cardiorespiratory dysfunction, and others that usually impact activities of daily living and quality of life.

The long-term sequelae of COVID-19 reaches general human physiological functions due to deregulated immune response during and after active infection. The greater focus of evidence of COVID-19 long-term sequelae in individuals who developed the severe form of COVID-19 is mainly cardiorespiratory, musculoskeletal, and psychological sequelae (1, 3). Studies have already shown that long-term sequelae can be found independently of the disease severity (4–6), justifying the importance of long-term monitoring of also the less severe cases that are often overlooked. Recent studies showed that PASC episodes with the dysfunctional immune response persist months after the primary infection. The possible mechanisms involved on immune-mediated PASC include an elevated concentration of inflammatory response markers, such as higher interleukin (IL)-6 and chemokines, as well as activated circulating T cells with upregulated exhaustion markers (i.e., programed death protein-1, PD-1) (7). Furthermore, cellular immunity is also dysregulated in PASC patients, with increased frequency of inflammatory non-classical CD14^+^CD16^+^ monocytes and a persistent overactive cytotoxic T- cell response, characterized by accumulation of terminally differentiated exhausted lymphocytes (8, 9), being observed. Thus, dysregulation in immune response may be associated with COVID-19 sequelae after mild to moderate infections.

Habitual practice of physical activity increases positive outcomes of COVID-19 (10, 11), also partly reducing the severity of SARS-CoV-2 acute manifestation (12). In a recent systematic review with non-linear dose–response meta-analysis involving 1,863,610 adults, Ezzatvar and colleagues (2022) demonstrated that regular physical activity is associated with a lower likelihood of adverse COVID-19 outcomes. Habitual physical activity may counteract the immune dysfunction status observed during and after SARS-CoV-2 infection due to the immunomodulatory role of exercise training (13). Chronic exercise decreases chronic inflammation and induces protection against the severity of acute pathogenic infections and chronic diseases (14). Additionally, regular physical activity improves the immune system and the response against infectious agents (15–18).

Additionally, habitual physical fitness is associated with a lower proportion of late-differentiated memory T cells and inflammatory CD16^+^ monocytes, concomitant with a higher frequency of naive T cells and immunoregulatory leukocytes (19–21). A pattern of habitual physical activity throughout the life cycle is essential for the balance between pro- and anti-inflammatory cytokines, hence the importance of lifelong exercise to stimulate an anti-inflammatory status (22). Thus, habitual physical activity immunoregulatory properties could attenuate immune dysfunction observed in post- COVID-19 conditions. However, much less is known regarding the role of exercise training and physical activity on post-viral sequelae conditions.

Here, we suggest that the physical activity level (PAL) can be associated with target outcomes of possible sequelae experienced in mild- to- moderate post-COVID-19. Therefore, the aim of this study was to evaluate if physical activity is associated with the inflammatory and molecular responses, as well as general clinical functions of individuals who recovered from mild- to- moderate COVID-19.

## Methods

### Study design and participants

This cross-sectional observational study is a part of the FIT-COVID Study research project detailed elsewhere (23). All included participants were aware of the study design and protocols and signed the informed consent form. The study was approved by the Ethical Institutional Review Board (approval number: 38701820.0.0000.5402) and registered on the Brazilian Clinical Trials Registry (registration number: RBR-5dqvkv3).

Men and women individuals (20–40 years) previously infected with mild-to-moderate COVID-19 (before vaccination), without comorbidities, were contacted to participate in the study. An age-matched healthy control group tested negative for SARS-CoV-2 (a test for IgM and IgG antibodies was conducted) was also recruited.

### Assessment schedules

#### General anamnesis

A general anamnesis was carried out including investigation of medication in use and self-reported COVID-19 symptoms (during acute infection and post- COVID-19 persistent symptoms).

#### Physical activity level

Physical activity (controlled and free-living environments) was estimated by triaxial accelerometers devices (GT3X+; ActiGraph, LLC, Pensacola, FL, USA). Sedentary time, light physical activity, and moderate–vigorous physical activity were defined according to Troiano et al. (24). Detailed methods can be found in our study protocol (23).

#### Anthropometric and body composition assessment

Stature and body weight were measured by a fixed stadiometer and an electronic scale, respectively. The body composition was estimated using DXA, a phase-sensitive bioimpedance analyzer, and ultrasonography. Detailed methods can be found in our study protocol (23).

#### Dietary intake

Nutritional information was recorded from 3-day food diaries (two weekdays and one weekend day). A nutritionist was responsible for instructing the participants to complete the food diaries. Total energy, protein, carbohydrates, and lipid intake were analyzed using NutWin software, version 1.5.

#### Pulmonary and peripheral muscle function

Pulmonary function was assessed by spirometry. Functional exercise capacity was assessed using the 6-minute walk test (6MWT). Muscle strength was estimated by sit-to-stand test (STS) and using electronic and mechanical dynamometers (handgrip and quadriceps femoris strength). Detailed methods can be found in our study protocol (23).

#### Mental health and quality of sleep

Mental health and quality of sleep were assessed using specific questionnaires of cognitive function, anxiety, depression, and sleep behavior. Detailed methods can be found in our study protocol (23).

### Blood collection and analysis

All participants visited the laboratory after a 12-h fasting period. Approximately 10 mL of blood from an antecubital vein was collected in a tube containing anticoagulant gel for serum isolation, ethylenediaminetetraacetic acid (EDTA) for plasma, and fluoride/EDTA for plasma to glucose analysis. The blood samples were centrifuged at 3,000 rpm for 15 min at 4°C, and the plasma and serum were stored at −80°C until further colorimetric and enzyme-linked immunosorbent assay (ELISA) analyses. A lateral flow test for IgM and IgG antibodies was performed using internal anti-SARSr-CoV IgG and IgM ELISA kits to detect previous SARS-CoV-2 infection.

Total cholesterol, triglyceride (TG), and glucose concentrations were analyzed using colorimetric kits (Labtest, Brazil) and insulin using ELISA commercial kits (Monobind Inc., USA). Homeostasis model assessment of insulin resistance (HOMA-IR) was calculated using the equation: HOMA-IR = (glucose [mmol/L] × insulin [μIU/mL]/22.5).

The concentrations of TNF-α, IL-6, IL-10, adiponectin, leptin, PAI-1, and BDNF were determined by ELISA with commercial kits (DuoSet R&D Systems, Minneapolis, USA) in the serum sample. The concentrations of TNF-α, IL-6, IL-10, and IFN-γ present in supernatants from PBMC cultures and stimulated whole blood were also determined by ELISA with commercial kits (DuoSet R&D Systems, Minneapolis, USA). The assays were performed following the manufacturer’s guidelines.

### Whole blood stimulated with LPS

We employed a protocol similar to that described by Barry et al. (25) for the whole blood- stimulated ex vivo assay. Approximately 3 mL of blood was collected in tubes containing K3-EDTA and diluted 1:10 in serum-free RPMI medium (Sigma) containing 5 mM glucose with 1× penicillin/streptomycin. Diluted whole blood was plated in 24-well culture plates (540 µL) and incubated in the presence or absence of lipopolysaccharide (LPS) (Escherichia coli, type: 0111: B4; Sigma, St. Louis, MO) at the final concentration of 10 ng/mL for 6 h at 37°C in 5% CO_2_. After this period, the supernatant was collected and stored at −80°C for further analysis.

### Peripheral blood mononuclear cell culture

The samples were added to Histopaque^®^-1077 (Sigma-Aldrich Co., LLC) (1:1) for PBMC isolation and centrifuged at 400 × g for 30 min at room temperature. The PBMC was washed with phosphate-buffered saline (PBS) and resuspended in 1 mL of enriched-medium RPMI. A total of 1 × 10^6^ PBMCs/mL were incubated for 24 h at 37°C and 5% CO_2_ in cell culture medium (RPMI-1640 Sigma-Aldrich Co., LLC) enriched with glutamine [2 mM], HEPES [20 mM], 10% fetal bovine serum, and antibiotics penicillin [100 U/mL] and streptomycin [0.1 mg/mL] in 24-well plates (Kasvi, PR, Brazil). PBMCs were cultured in the absence or presence of LPS [10 ng/mL] (Escherichia coli, type: 0111: B4; Sigma, St. Louis, MO) to measure innate inflammatory response, or with phorbol 12-myristate 13-acetate (PMA) [50 ng/mL] (Sigma, St. Louis, MO) plus ionomycin [1 μg/mL] (Sigma, St. Louis, MO) to verify adaptive cytokine production. After 24 h, supernatants were collected and stored at −80°C for further cytokine analysis.

### Cell staining and flow cytometry evaluation

The samples were added to Histopaque^®^-1077 (Sigma-Aldrich Co., LLC) (1:1) for PBMC isolation and centrifuged at 400 × g for 30 min at room temperature. PBMC viability was determined by trypan blue exclusion, and the viability was always more than 95%. Then, the cells were washed and suspended in RPMI‐1640 medium (Sigma‐Aldrich, USA) supplemented with 2 g/L sodium bicarbonate, 2% glutamine, and 100 U/mL penicillin – 0.1 mg/mL streptomycin (Sigma‐Aldrich, USA). Then, cells were washed with phosphate-saline buffer 1× and frozen at − 80°C in a solution containing 90% fetal bovine serum and 10% dimethyl sulfoxide (DMSO) until the day of analysis.

Cells were thawed by diluting them in 5-mL pre-warmed complete RPMI 1640 medium (Sigma-Aldrich —R8758) containing 5% FBS and spun at 1,500 rpm for 5 min. Supernatants were carefully removed, and cells were resuspended in RPMI 1640 medium. The viability of cells (>98%) was examined using trypan-blue staining (Gibco, Grand Island, New York, USA). Briefly, 2 × 10^5^ PBMCs were stained with monoclonal antibodies (all anti-human) conjugated with the following specific fluorochromes: CD4 FITC (Clone OKT-4), CD8 Pe (Clone RPA-T8), CD25 Pe (Clone BC 96), CD127 Percp-Cy5.5 (Clone eBioRDR5, CD28 Percp-Cy5.5 (Clone CD28.2), PD-1 APC (Clone MIH 4), CD14 FITC (Clone 61D3), CD16 Pe (Clone eBioCB16), HLA-DR Percp-Cy7 (Clone G46-6) (all from Invitrogen, USA). Cell phenotype was acquired using CellQuest Pro Software (BD Bioscience, USA) on a FACSCalibur flow cytometer (BD Biosciences, USA). A minimal of 20,000 events/tubes were acquired, and lymphocytes were identified and gated according to each forward scatter (FSC) and side scatter (SSC) profile. The mean fluorescence intensity (MFI) of CD28 and PD-1 was analyzed in CD4+ and CD8+ T- cell subpopulations, and HLA-DR expression was evaluated in CD14+CD16− and CD14+CD16+ monocyte subsets. The Treg phenotype was defined as CD4+CD25^high^CD127^low^ according to Liu et al. (26).

### Measurement of high-resolution respirometry in permeabilized PBMC

A total of 0.5 × 10^6^ PBMCs were utilized to measure mitochondrial respiration. Respiration of permeabilized cells was performed using the protocol described and developed by Oroboros (Oroboros Instruments, Innsbruck, Austria) with modifications (27, 28). ROUTINE respiration which represents the consumption of mitochondrial O_2_ under endogenous conditions was measured from the addition of cells to the respiration medium (MiR05). The complex I (CI) LEAK respiration was measured with the addition of malate (5 mM), glutamate (10 mM), and pyruvate (5 mM), which represents mitochondrial O_2_ consumption after inhibition of ATP synthesis compensating for proton leak. The oxidative phosphorylation (OXPHOS) was obtained first by ADP (2.5 mM) addition (OXPHOS CI) followed by succinate (10 mM) (OXPHOS CI+CII) to obtain the maximal respiratory rate, reflecting the maximum capacity of mitochondrial producing ATP from oxidative phosphorylation (29). All measurements were recorded at 37°C.

### RNA isolation and RT-PCR assays

Total RNA from PBMC was extracted with Brazil reagent (LGC Biotechnology Ltda., Cotia, SP) according to the manufacturer’s recommendations and was used for the RT-PCR analyses. Reverse transcription to complementary DNA (cDNA) was performed using the High-Capacity cDNA Reverse Transcription kit (Applied Biosystems, Thermo Fisher Scientific, Foster, CA). The cDNA was stored at −80° C for subsequent analysis (AMPK, NF-kB, HIF-1α, TLR-4, AR-β1, AR-β2, BMAL1, REV-ERBα) by RT-PCR with Power SYBR Green PCR Master Mix (Applied Biosystems). Primer sequences are presented in **Supplementary Table 1**. Quantification of gene expression was carried out using the glyceraldehyde-3‐phosphate dehydrogenase gene (GAPDH) and β-tubulin as an internal control. Relative quantification of genes of interest was calculated using the 2−ΔΔCT formula, in which cycle threshold (CT) is the difference between the CT value for the gene of interest and the CT value for the GAPDH as housekeeping gene.

### Statistical analysis

Statistical procedures were performed using SPSS (version 22.0). Data distribution was analyzed using the Shapiro–Wilk test. For clinical, systemic blood parameters and cellular phenotype comparisons between the post-COVID-19 and control groups, the unpaired t-test or Mann–Whitney U test were used according to data distribution. The Fisher test was performed for categorical variables. For cellular stimuli analysis, a two-way analysis of variance was used to compare group, time, and interaction group × time. Effect sizes (ESs) were calculated for significant secondary comparisons and classified as negligible (<0.01), small (0.1–0.29), medium (0.3–0.49), and large (>0.5). Secondarily, the data were analyzed using analysis of covariance (ANCOVA) to better understand the associations of PA on the study data (30). The dependent variable was represented by each clinical, systemic blood parameters and cellular data, and MVPA was used as the covariate in this multivariable regression. Correlational analysis between clinical and immuno/biochemical data in the post-COVID-19 group was performed using Pearson or Spearman test according to data distribution.

## Results

Participants of both sexes with mild to moderate clinical COVID-19 infection were recruited after 70.50 ± 43.10 days of diagnosis. A healthy age-matched, control group that was negative for SARS-CoV-2 was also recruited (**Table 1**). No differences in sex distribution and anthropometric characteristics were observed between groups.

**Table 1:** General characteristics of participants.

	N	Control	N	Post-COVID-19	*p* value	*p* adjusted
Gender						
Men, N (%)		14 (70)		11 (55)	0.327	–
Women, N (%)		6 (30)		9 (45)		
Anthropometry						
Age (y)	**20**	29.39 (21.25 – 32.62)	**20**	29.41 (21.90– 34.96)	0.620	0.727
Body weight (kg)	**20**	77.50 ± 19.21	**20**	76.48 ± 14.42	0.850	0.746
BMI (kg m^2^)	**20**	25.46 ± 4.78	**20**	25.51 ± 4.32	0.971	0.993
Waist circumference (cm)	**20**	85.93 ± 14.35	**20**	87.69 ± 11.34	0.669	0.862
Body composition						
Total lean mass (kg)	**20**	51.43 ± 13.39	**19**	48.66 ± 10.51	0.479	0.140
Total lean mass (%)	**20**	67.56 ± 8.49	**19**	64.45 ± 10.60	0.316	0.084
Body fat (kg)	**20**	18.60 (14.00– 28.75)	**19**	22.75 (16.10 – 32.66)	0.428	0.518
Body fat (%)	**20**	28.61 ± 8.85	**19**	31.91 ± 11.06	0.310	0.086
VAT (cm)	**20**	3.20 (2.52 – 3.95)	**20**	3.10 (2.02– 4.32)	0.678	0.899
SAT (cm)	**20**	1.35 (1.12 – 2.07)	**20**	1.55 (1.00– 2.62)	0.947	0.995
Steatosis (no): no/yes	**20**	18/2	**20**	17/3	0.063	–
Steatosis grade: N (%)	**20**		**20**			
0		18 (90.00)		17 (85.5)		
1		1 (5.0)		3 (15.00)		
2		1 (5.0)		0 (0)		
Physical activity						
LPA (min day^−1^)	**20**	272.00 ± 69.69	**20**	290.88 ± 93.96	0.602	0.040
MPA (min day^−1^)	**20**	24.58(13.39 – 49.39)	**20**	13.35 (8.70 – 24.29)	0.102	<0.001
VPA (min day^−1^)	**20**	2.39 (0.00 – 11.82)	**20**	0.00 (0.00 – 2.85)	0.072	<0.001
MVPA (min day^−1^)	**20**	30.35 (13.68 –61.56)	**20**	13.57 (9.38 –27.25)	**0.038**	**-**
Sedentary behavior (h day^−1^)	**20**	9.37 (8.56 –10.35)	**20**	8.49 (8.03– 9.62)	0.121	0.353

BMI, body mass index; VAT, visceral adipose tissue; SAT, subcutaneous adipose tissue; LPA, light physical activity; MPA, moderate physical activity; VPA, vigorous physical activity; MVPA, moderate to vigorous physical activity. Bold values p < 0.05 compared with control. p adjusted: between- group comparisons were performed using analysis of covariance (ANCOVA) with adjustment for MVPA. Data are presented as mean ± SD for data with normal distribution and median (IQR) for data with non-normal distribution.

### Clinical outcomes

The groups had a similar body composition (**Table 1**). The post-COVID-19 group presented lower values of moderate to vigorous physical activity (MVPA) (ES = 0.54) compared with the control group (**Table 1**). For this reason, all study comparisons were also performed with adjustment for MVPA to analyze the association of physical activity level in the results.

Daily food consumption was also similar between the control and post-COVID-19 groups (*data not shown*, p>0.05); however, when the analysis was adjusted for MVPA, daily protein consumption was significantly lower in the post-COVID-19 group (CTL: 92.59 g (64.99 – 138.45) vs post-COVID-19: 87.37 g (64.76 – 122.78) adj p = 0.018; adj ES = 0.24). Participants previously infected with COVID-19 performed a shorter distance in the 6-minute walk test (ES = 0.65; **Table 2**), but this result was due to the influence of the level of physical activity also lower in the post-COVID group, as evidenced by the loss of significance after adjustment by MVPA (**Table 2**). Regarding the pulmonary function parameters evaluated, the post-COVID-19 group presented lower values of FEV_1_ (ES = 0.84), FEV_1%_ pred (ES = 0.75), FVC (ES = 0.85), and FVC % pred (ES = 0.80) compared with the control (**Table 2**).

**Table 2:** Muscular and pulmonary function of participants.

	N	Control	N	Post-COVID-19	*p* value	*p* adjusted
Muscular function						
Handgrip (kgf)	**20**	42.02 ± 11.30	**20**	38.08 ± 12.84	0.309	0.250
Sit-and-stand test (s)	**20**	10.05 ± 2.48	**20**	9.24 ± 1.93	0.260	0.426
6MWT (meters)	**20**	682.10 ± 74.43	**20**	630.50 ± 83.69	**0.046**	0.109
Quadriceps femoris strength (kg)	**14**	95.50 ± 50.50	**17**	97.17 ± 40.09	0.910	0.424
Pulmonary function						
Respiratory rate (bpm)	**20**	16 (16 – 16)	**20**	16 (16 – 16)	0.640	0.710
SpO_2_ (%)	**20**	98 (97 – 98)	**20**	98 (98 – 98)	0.698	0.777
FEV1 (L)	**20**	3.64 ± 0.79	**20**	2.98 ± 0.78	**0.012**	**<0.001**
FEV1% predicted	**20**	87.95 ± 11.44	**20**	76.60 ± 18.07	**0.024**	**0.008**
FVC (L)	**20**	4.29 ± 1.06	**20**	3.43 ± 0.96	**0.010**	**<0.001**
FVC % predicted	**20**	86 (75.00 – 97.00)	**20**	74.40 (60.00 – 91.00)	**0.026**	**<0.001**
FEV1/FVC (%)	**20**	86.20 (83.00 – 92.00)	**20**	85.70 (83.00 – 86.50)	0.369	0.669

6MWT, six-minute walk test; SpO_2_, peripheral oxygen saturation; FVC, forced vital capacity; FEV1, forced expiratory volume in one second. Bold values p < 0.05 compared with control. p adjusted: between group comparisons were performed using analysis of covariance (ANCOVA) with adjustment for MVPA. Data are presented as mean ± SD for data with normal distribution and median (IQR) for data with non-normal distribution.

The results of clinical parameters during the six-minute walk test (after 3 min and after 6 min) and at test recovery between control and post-COVID-19 subjects are illustrated in **Supplementary Figure 1**. The scores of lower-limb RPE scale (ES: 0.65, adj ES: 0.33) (**Supplementary Figure 1A**), perception of dyspnea (ES: 0.44, adj ES: 0.43) (**Supplementary Figure 1B**), and SatO_2_ ES: 0.24, adj ES: 0.16) (**Supplementary Figure 1D**) were changed over time (after 3 min and 6 min) returning to resting values at recovery, with no differences between groups regardless of MVPA adjustment. Similarly, heart rate (ES: 0.59, adj ES: 0.46) (**Supplementary Figure 1C**) and respiratory rate (ES: 0.50, adj ES: 0.35) (**Supplementary Figure 1E**) were also changed over time between the control and post-COVID-19 groups, even after adjusting for physical activity level. However, those parameters did not return to baseline values after recovery.

Mental health (anxiety, depression, memory, and attention) and sleep quality were evaluated. The results are shown in **Supplementary Table 2**. All participants had a sleep quality mean total score >5, with no difference between the control and post-COVID-19 groups, even after adjustment for MVPA. The degree of daytime sleepiness, as measured by the Epworth sleepiness scale, was also similar between groups, even after adjustment for MVPA. In detail, both groups had a total score < 10; values above this cutoff point correspond to a high probability of daytime sleepiness. Mental health was also assessed using cognitive function, anxiety, and depression questionnaires. The total scores for anxiety and depression symptoms (measured by the HADS scale), and the intensity of depression symptoms (assessed by the Brazilian version of the Beck Depression Inventory), were similar between the groups (p > 0.05). In the HADS scale, the recommended cutoff scores were used, which are < 7 for unlikely cases, 8–11 for doubtful cases, and ≥ 12 for probable cases. The results showed that both groups were classified as doubtful cases for symptoms of anxiety and depression. The scores of the Brazilian version of the Beck Depression Inventory range from 0 to 63 points (0–11 minimal or normal; 12–19 mild; 20–35 moderate; and 36–63 severe). The intensity of depression symptoms was considered normal for all participants, even after adjustment for MVPA. The digit span test evaluated memory and attention capacity, and the mental manipulation of information in two steps. Both conditions require participants to verbally repeat the requested sequences in forward or reverse order. The straight-order sequence score was lower in the post-COVID-19 group compared with the control group (ES: 0.81); however, when adjusted for MVPA, the groups had similar results.

### Immunometabolic outcomes

**Figure 1:**
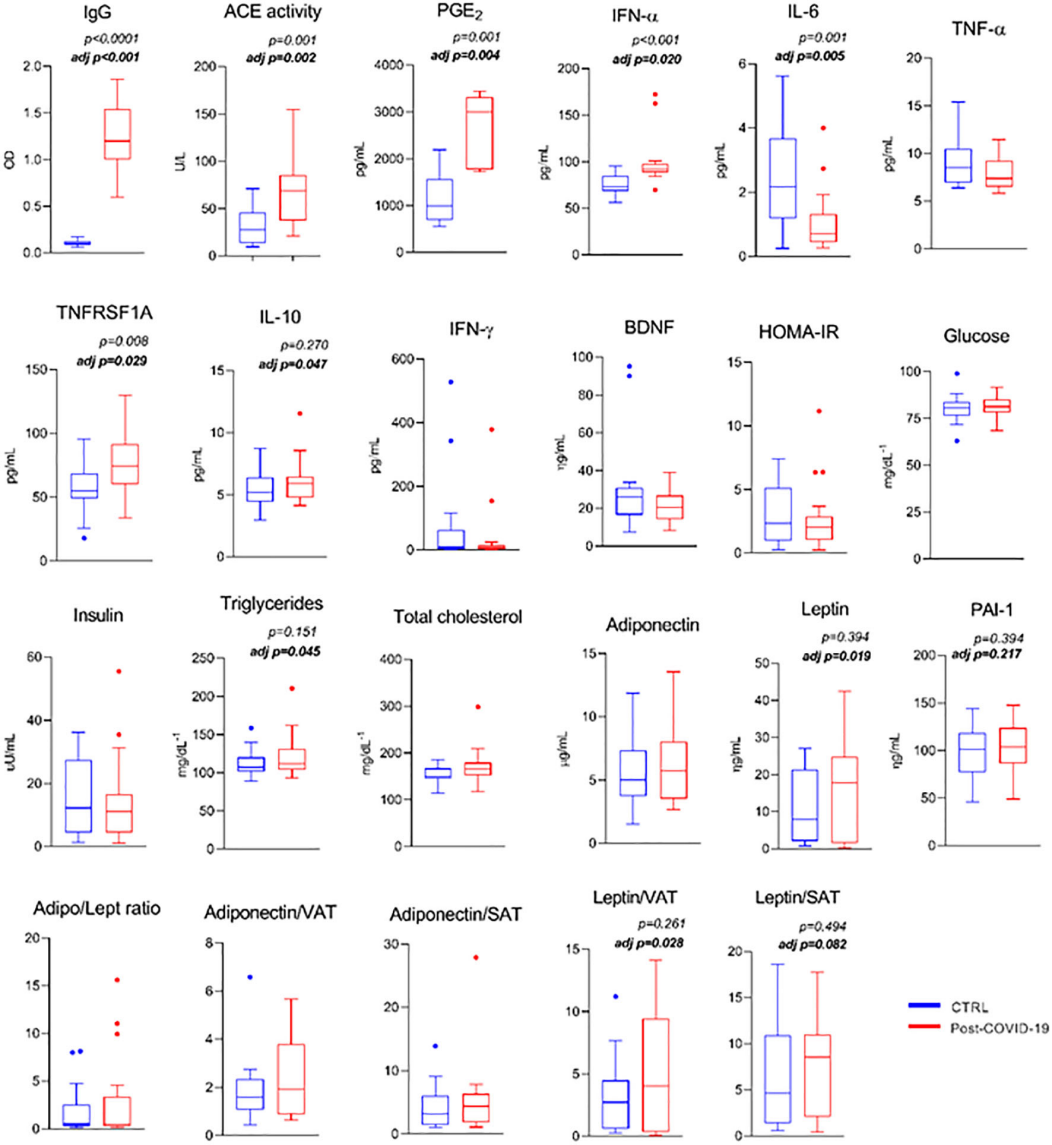
Serum metabolic markers and pro- and anti- inflammatory response between control and post-COVID-19. Values expressed as mean ± SE of IgG (OD) (control n = 14; COVID-19 n = 17); Ace activity (U/L) (control n = 14; COVID-19 n = 18); PGE-2 (control n = 9; COVID-19 n = 5); INF-α (pg/mL); IL-6 (pg/mL); TNF- α (pg/mL); TNFRSF1A (pg/mL); IL-10 (pg/mL); IFN-γ (pg/mL); BDNF (ng/mL); HOMA-IR; glucose (mg/dL^−1^); insulin (μU/mL); triglycerides (mg/dL^−1^); total cholesterol (mg/dL^−1^); adiponectin (μg/mL); leptin (μg/mL) (control n = 20; COVID-19 n = 20) adj p: between- group comparisons were performed using analysis of covariance (ANCOVA) with adjustment for MVPA. p value set < 0.05.

Comparison of serum metabolic and pro- and anti-inflammatory markers is shown in **Figure 1**. The post-COVID-19 group showed a higher serum concentration of SARS-CoV-2 IgG titers (ES: −3.96), increased ACE activity (ES: −1.27), PGE- 2 (ES: −2.35), and higher INF-α (innate antiviral defense; ES: −1.13) and soluble TNF-α receptor (anti-inflammatory; ES: −0.92) levels compared with the control group, even after adjustment for MVPA (ES: 0.80; ES: 0.34; ES: 0.62; ES: 0.25, ES: 0.18, respectively). The same group presented a lower serum concentration of IL-6 (ES: 1.12) compared with the control group, even after adjustment (ES: 0.24). When adjusted for physical activity level, the group with a previous episode of mild to moderate COVID-19 infection showed a higher triacylglycerol level (ES: 0.18), larger content of the anti-inflammatory cytokine IL-10 (ES: 0.45), leptin level (ES: 0.19), leptin/visceral adipose tissue ratio (ES: 0.17), and leptin/subcutaneous adipose tissue depot (ES: 0.12) (trend) compared with the control group (**Figure 1**). Also, comparison of serum metabolic and pro- and anti-inflammatory markers between groups was performed according to fat mass (%) see **Supplementary Figure 2**.

**Figure 2:**
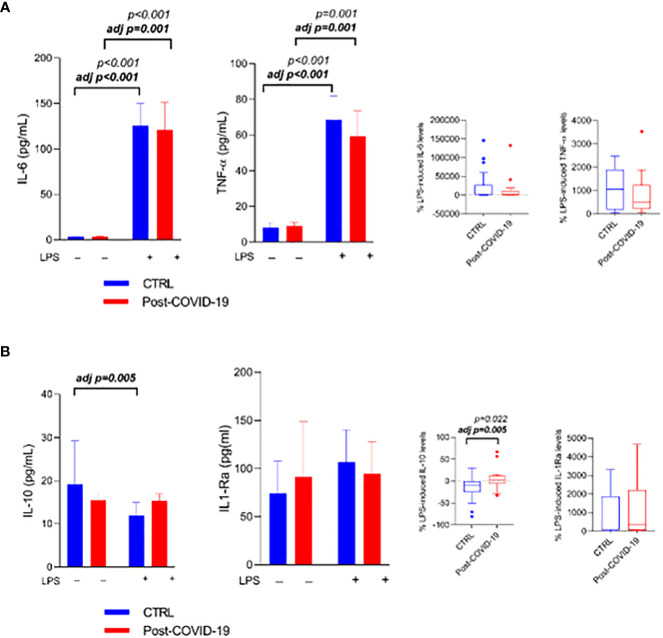
Whole blood stimulated in the presence or absence of LPS [10 ng/mL) between control and post-COVID-19. Values expressed as mean ± SE of **(A)** IL-6 (pg/mL); TNF- α (pg/mL) and **(B)** IL-10 (pg/mL) IL1-Ra (pg/mL) (control n = 20; COVID-19 n = 20). The percent change of IL-6, TNF- α **(A)** and IL-10; IL1-Ra **(B)** were compared for both stimulated conditions. adj p: between- group comparisons were performed using analysis of covariance (ANCOVA) with adjustment for MVPA. p value set < 0.05.

When participants’ whole blood was stimulated in the absence (control) and presence of LPS (LPS) (**Figure 2**), the inflammatory stimulus increased IL-6 production (stimulus; ES = CTL: 0.32; COVID: 0.29), adjusted by MVPA (ES = CTL: 0.33; COVID: 0.27) (**Figure 2A**), and TNF-α (ES = CTL: 0.34; COVID: 0.26), adjusted by MVPA (ES = CTL: 0.33; COVID: 0.24) (**Figure 2A**) in both groups. In relation to anti-inflammatory cytokines, IL-10 production decreased only in the control group after adjustment by MVPA (adjusted by MVPA interaction time × group p = 0.02, time p < 0.01, ES: 0.20; **Figure 2B**), with a difference between the relative production of IL-10 between the groups (ES = -0.75; adjusted by MVPA ES: 0.25 **Figure 2B**), and there was no change in the production of IL-1ra (**Figure 2B**) in both groups.

**Figure 3:**
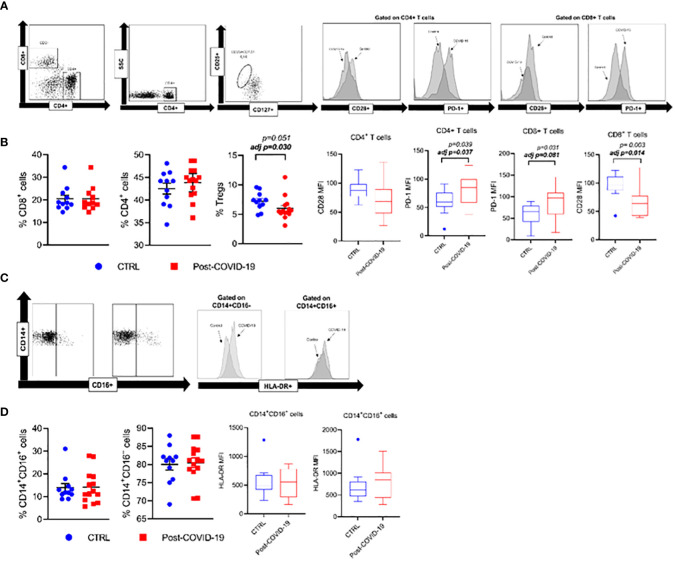
Phenotypic expression of lymphocyte and monocytes subpopulations, and cellular checkpoint markers in control and Post-COVID-19. **(A)** Strategy gate of CD4+ and CD8+ T cells **(B)** CD3+CD4+, CD3+CD4+, CD3+CD8+ and Treg cells %, CD3+CD4+ CD28+, CD3+CD8+CD28+, CD3+CD4+ PD-1+, CD3+CD8+ PD-1+ **(C)** Strategy gate of CD14+CD16-, CD14+CD16+ cells **(D)** CD14+CD16-, CD14+CD16+ %, CD14+CD16-HLA-DR+, CD14+CD16+HLA-DR+ (Control n=11; COVID-19 n=14). Data are presented as mean ± SE. adj p: between group comparisons were performed using analysis of covariance (ANCOVA) with adjustment for MVPA. P value set < 0.05.

Phenotypic expressions of lymphocyte and monocyte subpopulations and cell stimulatory/inhibitory checkpoint markers in control and post-COVID-19 are illustrated in **Figure 3**. Post- COVID-19 individuals exhibit a lower percentage of Treg compared with control. Additionally, CD4^+^ and CD8^+^ T cells from subjects who had COVID-19 exhibit a higher expression of the programmed cell death marker PD1 when compared with control subjects (**Figure 3B**). Also, CD8^+^ T cells from individuals who had previous COVID-19 showed a lower expression of the co-stimulatory molecule CD28 compared with control. There were no significant differences between the phenotypic expression of monocytes between the groups (**Figures 3C, D**).

**Figure 4:**
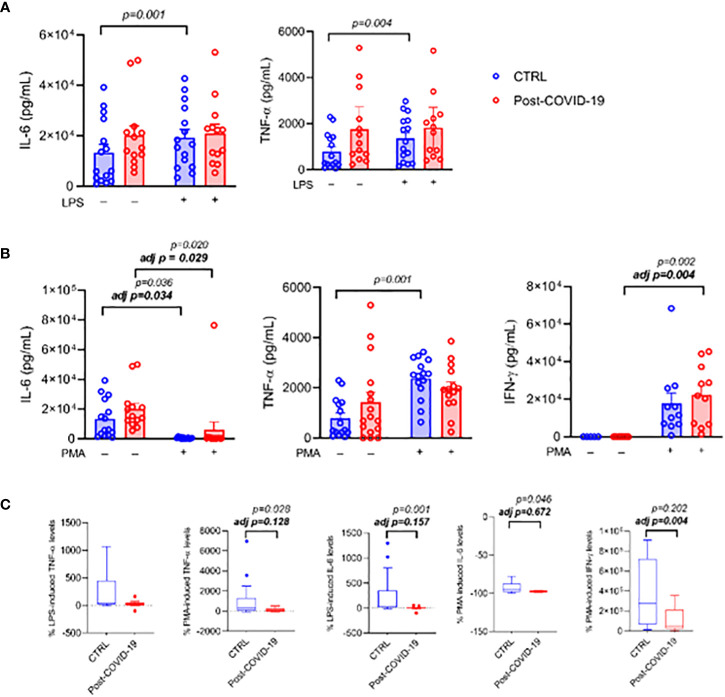
PBMC cultured in the presence or absence of LPS [10 ng/mL] or PMA [50 ng/mL] plus Ionomycin [1 μg/mL] between control and post-COVID-19. Values expressed as mean ± SE of **(A)** IL-6 (pg/mL); TNF- α (pg/mL) (control n = 15; COVID-19 n = 13) for control and LPS-stimulated condition and **(B)** IL-6 (pg/mL) (control n = 15; COVID-19 n = 13); TNF- α (pg/mL) (control n = 15; COVID-19 n = 16); IFN-γ (pg/mL) (control n = 11; COVID-19 n = 11) for control and PMA+ ionomycin-stimulated conditions. The percent change of **(C)** IL-6, TNF- α, and IFN-γ were compared for both stimulated conditions. adj p: between- group comparisons were performed using analysis of covariance (ANCOVA) with adjustment for MVPA. p value set < 0.05.

When PBMCs were cultured and stimulated with LPS (**Figure 4**), the inflammatory stimulus promoted an increase in IL-6 production only in the control group (interaction group × time p = 0.03, time p < 0.01, ES: 0.37); however, after adjustment by MVPA, there was no longer a change under stimulus (time p = 0.40, **Figure 4A**). A similar response occurred with TNF-α (time p < 0.01, ES: 0.31, adjusted by MVPA: time p = 0.06, **Figure 4A**). After stimulation of PBMCs with PMA plus ionomycin, there was an increase in IL-6 production in both groups (time p < 0.01, ES = CTL: 0.32; COVID: 0.29; adjusted by MVPA time p = 0.04, ES = CTL: 0.33; COVID: 0.27 **Figure 4B**). TNF- α production increased only in the control group (time p = 0.01, interaction time × group p = 0.03, ES = 0.35); after adjustment by MVPA, no change under stimulus was detected (time p = 0.25, **Figure 4B**). On the other hand, stimulation with PMA plus ionomycin caused an increase in IFN-γ production only in the post-COVID-19 group (time p < 0.01 ES = 0.63; adjusted by MVPA time p = 0.02 ES = 0.62 **Figure 4B**). When the relative production of cytokines was compared between the post-COVID-19 control groups, the post-COVID-19 group showed lower relative production of IL-6 (ES = 0.35 **Figure 4C**) and of TNF-α (ES = 0.73 **Figure 4C**) when cells were stimulated with PMA plus ionomycin; however when adjusted for MVPA, the differences disappeared. The production of IFN-γ was lower in the post-COVID-19 group only after adjustment by MVPA (ES = 0.20). On the other hand, when PBMCs were stimulated with LPS, the relative production of IL-6 cytokines was lower in the post-COVID-19 group (ES = 0.75) and TNF-α was not different between the groups (**Figure 4C**).

**Figure 5:**
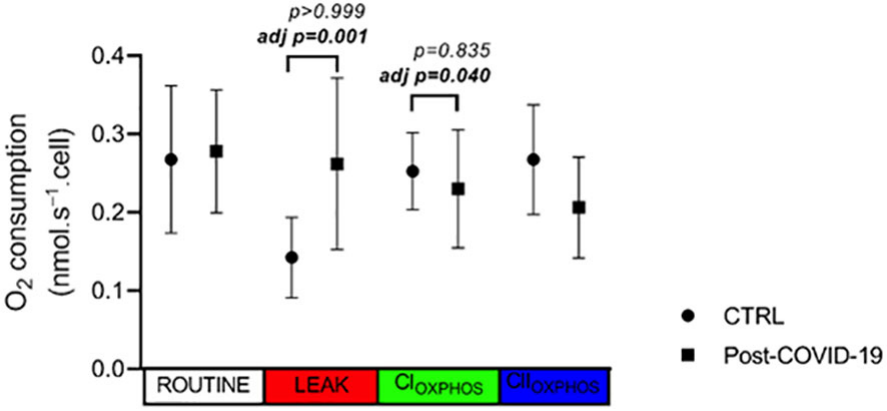
PBMC oxygen consumption rate in different status of mitochondrial respiration in control and post-COVID-19. Values expressed as mean ± SE (control n = 4; COVID-19 n = 5). adj p: between- group comparisons were performed using analysis of covariance (ANCOVA) with adjustment for MVPA. p value set < 0.05.

Mitochondrial respiration profiles of PBMCs are shown in **Figure 5**. No differences in O_2_ consumption rates were observed in ROUTINE, LEAK, OXPHOS(CI), and OXPHOS(CI+CII) states in PBMCs between the post-COVID-19 and control groups. However, after adjustment by MPVA, we observed a higher value in the LEAK state and lower OXPHOS(CI) in the post-COVID group compared with the control (**Figure 5**). Thus, mild to moderate COVID-19 appears to have a direct impact on mitochondrial respiration of PBMCs.

**Figure 6:**
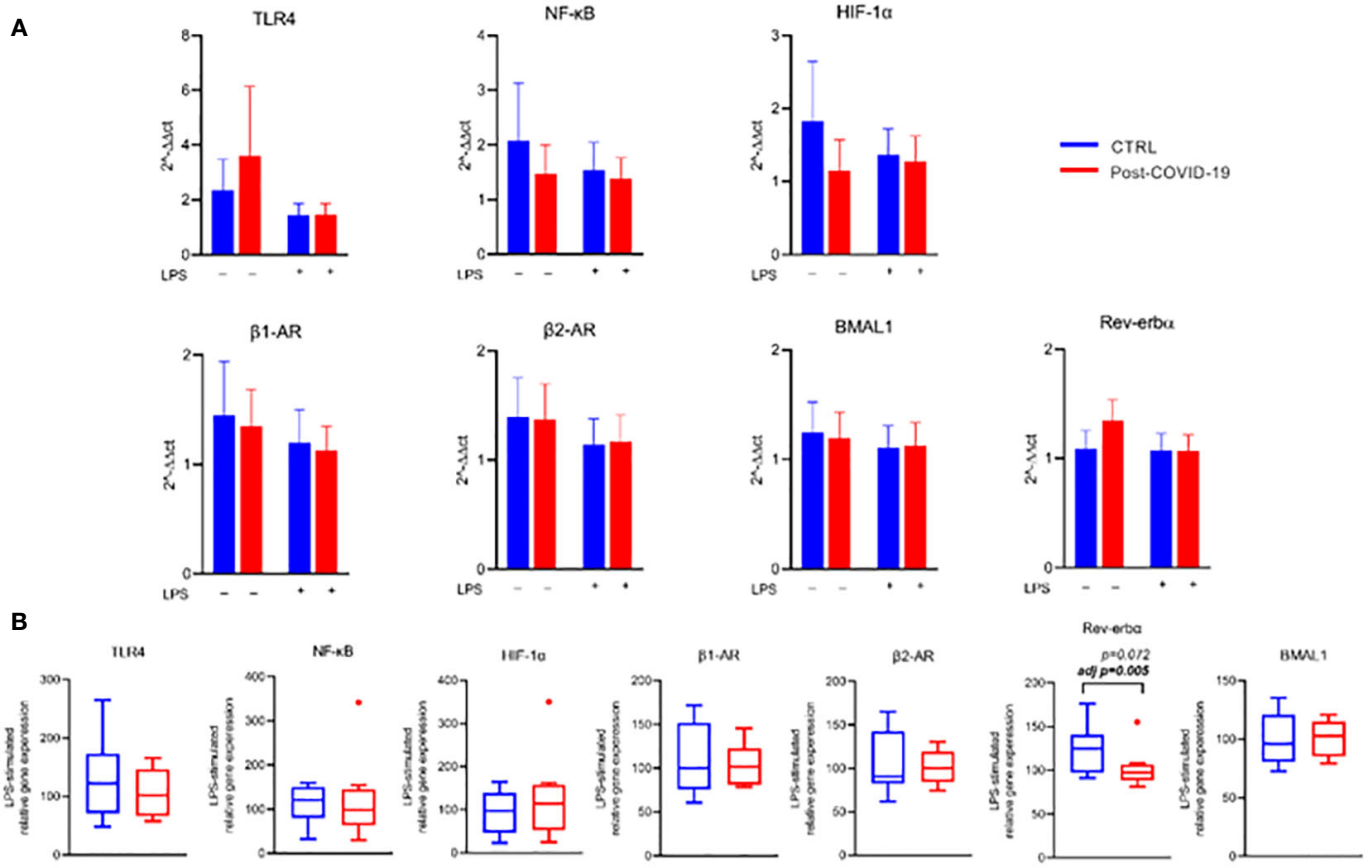
Gene expression relative to B-tubulin in PBMC stimulated in the absence or presence of LPS [10 ng/mL] between control and post-COVID-19. Values expressed as mean ± SE of **(A)** TLR-4 expression, NF-κB expression, HIF-1α expression, α1 receptor expression, α2 receptor expression, BMAL1 expression, Rev-Erb-α expression (2^−ΔΔct^). **(B)** Respective genes percent changes for both stimulated condition (control n = 8; COVID-19 n = 9). adj p: between- group comparisons were performed using analysis of covariance (ANCOVA) with adjustment for MVPA. p value set < 0.05.

In relation to the expression of genes related to inflammatory pathways, beta adrenergic receptors, and specific clock genes in PBMCs (**Figure 6**, **Supplementary Figure 3**), we did not observe significant changes after stimulation with PMA and LPS compared with untreated cells, regardless of the group and adjustment by MVPA. Only after MVPA adjustment was there a lower relative and absolute expression of the Rev-Erb-α clock gene after LPS stimulation in the post-COVID-19 group compared with the control group (**Figure 6B**). Such behavior reflects a punctual effect of COVID-19 on the modulation of the Rev-Erb-α clock gene, an integrator of circadian rhythms and metabolism.

The production of IL-6 and TNF-α in lymphocytes cultured with PMA plus Ionomycin and in monocytes cultured with LPS in the control and post-COVID-19 groups is shown in **Supplementary Figure 4**. Stimulation of monocytes with LPS did not increase IL-6 and TNF-α production in both groups (**Supplementary Figure 4A**). After stimulation of lymphocytes with PMA plus ionomycin, there was an increase in TNF-α production in both groups (ES = CTL:0.47; COVID: 0.34; adjusted by MVPA ES = CTL: 0.45; COVID: 0.34, with no effect on IL-6 production (**Supplementary Figure 4B**).

**Figure 7:**
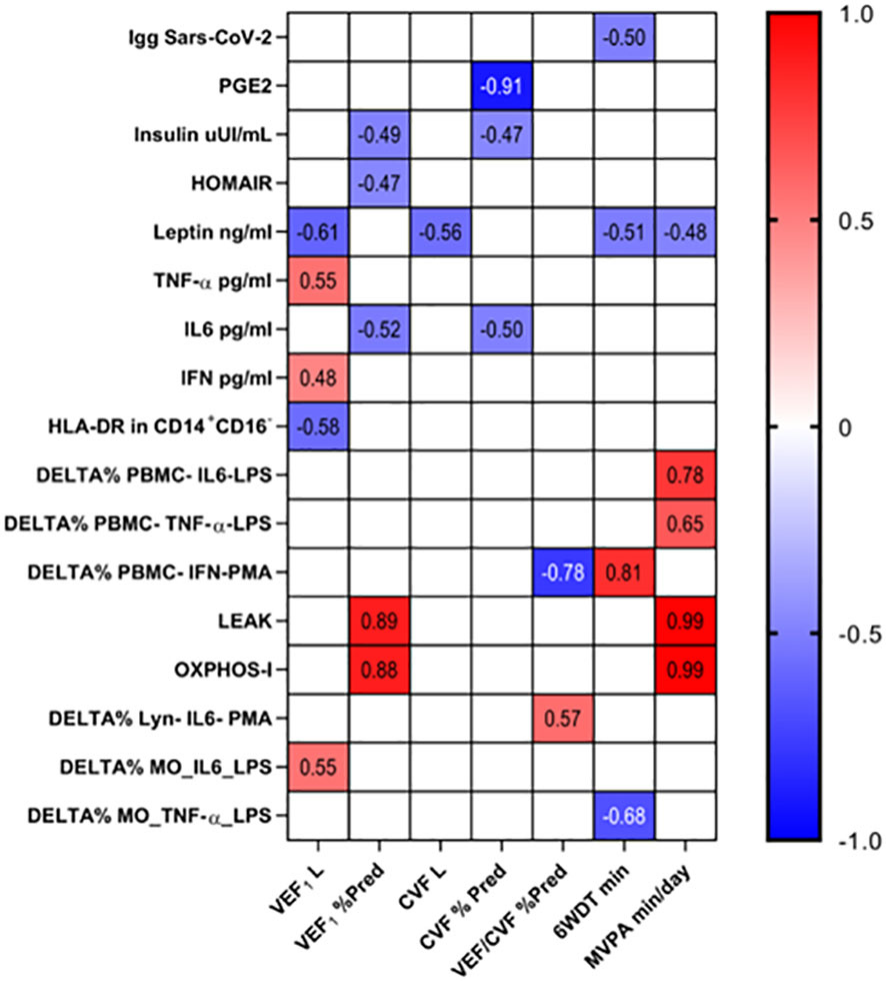
The association of clinical and immuno/biochemical data. The correlation between clinical and immuno/biochemical data were performed by Pearson’s coefficient correlation test and the significant results were presented (p < 0.05).

Finally, we correlated the clinical variables (which showed a significant difference between the control and post-COVID-19 groups) with the immuno/biochemical data studied (**Figure 7**). Moderate to very strong correlations were found among the variables studied showing associations between lung function and physical activity level with immuno/biochemical characteristics even in subjects who had mild to moderate COVID-19 (**Figure 7**).

## Discussion

Patients after mild to moderate SARS-CoV-2 infection exhibited important changes in functional, metabolic, and immune outcomes. An interesting point, our study engaged only subjects who were healthy, without chronic disease, presenting a past history of SARS-CoV-2 infection with outpatient/community treatment. On the other hand, the physical activity level appeared to confer a partial association in several parameters evaluated in the present study.

Our study showed that even mild to moderate cases without comorbidities result in a decrease in MVPA, with only 25% of post-COVID-19 individuals with sufficient physical activity (>150 min of MVPA) against 65% of the control group (p = 0.01). Delbressine et al. (2021) demonstrated that participants with persistent symptoms post-COVID-19 still presented reduced self-reported physical activity, 6 months after acute infection. The authors reported that 40.6% of the individuals included in the study had comorbidities and 25.9% were hospitalized in the acute phase of the disease (31).

The prevalence of physical and pulmonary function impairment after different clinical courses of COVID-19 remains inconstant. We demonstrate lower lung volumes (FVC and FEV1) in patients mild to moderate post-COVID-19, as commonly found in those patients who have had the severe disease (13). A subgroup of COVID-19 survivors demonstrated persistent lung parenchymal impairments, including pulmonary fibrosis (32). Guler et al. (2021) suggest that this process is due to a lung parenchymal issue rather than a respiratory muscle issue due to the maintenance of respiratory muscle strength, even after severe COVID-19. Although the pulmonary function can be influenced, among other factors, by the physical conditioning, in our study the differences remained after adjustment by MVPA, evidencing the influence of COVID-19 on pulmonary function in these patients (33).

We observed a significant difference for 6MWT finding between control and COVID-19 groups, with no difference when adjusted by MVPA. In this sense, the time spent in MVPA is associated with muscle function independently of mild-COVID-19 symptoms in patients non-hospitalized. This result was expected given the known positive influence of physical activity in functional capacity (34) and our results for distance performed in the 6MWT and the MVPA time being lower in the post-COVID-19 group compared with the control group.

### Systemic alteration of metabolic and inflammatory parameters

PASC, as well as other post-viral syndromes, is now recognized as a condition caused by deregulated immune response during and after active infection. Here, we found that post-COVID-19 patients presented higher systemic levels of PGE_2_, TNFRSF-1a, and IFN-α concomitantly to lower IL-6 concentrations. Our results may indicate that post COVID-19 is characterized by a state of immunosuppression and increased antiviral cytokines that remained for at least 4 months. Previous studies found increased the Th2 cytokine profile (i.e., IL-4 and IL-15) and IL-10 in the blood of recovered mild COVID-19 patients without sequelae (35, 36). On the other hand, higher IL-6 levels were previously related to debilitating symptoms on post- COVID-9 conditions, with PASC patients presenting higher IL-6 and TNF-α concentrations compared with non-PASC and healthy controls (37–39). Interestingly, post- COVID-19 patients of our study were mostly healthy and reported very few debilitating symptoms, demonstrating lower IL-6 levels.

Collectively, the lower IL-6 levels associated with higher PGE_2_ and TNFRSF1a levels suggest that non-PASC patients may present a diminished inflammatory response instead of increased classical cytokines related to low-grade inflammation. Conversely, antiviral cytokines remain higher after viral infections as a trained immunity mechanism to counteract persistent viral peptides that remains in the tissues months after original infections. Corroborating with this hypothesis, Klein and coworkers identified antiviral reactivity patterns maintained in long COVID-19 patients persisting in association with the reactivation of latent virus (40). In a cohort study with long COVID-19 and non-long COVID-19 patients, Phetsouphanh and colleagues (2022) identified higher IFN type I (IFN-α and IFN-β) 8 months after SARS-CoV-2 infection (38). Type I and III IFN signaling produced by murine lung dendritic cells in response to synthetic viral RNA is associated with damage to the lung epithelium and hampered lung repair during influenza infection in mice (41). In this sense, it was possible that the poor performance in respiratory parameters observed in our cohort study may be associated with a persistence of antiviral inflammatory response after COVID-19.

PGE_2_ increases during acute SARS-CoV-2 infection in a severity dependence (42, 43), impairing B-cell- mediated immune response and Th1 polarization by inhibiting myeloid cell function (44). The production of PGE_2_ by macrophages remains higher after influenza A virus infection episodes, although current reports did not indicate that the same occurs after SARS-CoV-2 infection (45). Thus, it was possible that post SARS-CoV-2 infection is characterized by non-inflammatory mediators that may hamper the immune response. To test this hypothesis, we exposed whole blood and PBMC to LPS (innate stimulation) and PMA (adaptive stimulation) to analyze cytokine production. Interestingly, the post- COVID-19 cohort presented lower IL-6, IFN-γ, and TNF-α production after cell culture incubation with pathogenic stimulus. These results reinforce the notion that SARS-CoV-2 may induce at least a transitory immunosuppression state characterized by diminished cytokine production after cell stimulation (46). Dysfunction of myeloid cells was previously reported in post- COVID-19 patients, as identified by a lower activation and homing markers that may indicate low antigen recognition and response (47). Also, LPS induced higher IL-10 production in post-COVID-19 patients, possible that it may be an attempt to overcome potential IL-10 resistance in this population (48). The greater IL-10 secretion in response to LPS may contribute to the drastic elevations in IL-10 seen in COVID-19 (49). In addition, cytokines related to adaptive immune response are altered in association with changes in the T- cell subset compartment (50).

Furthermore, chronic type I IFN signaling expands PD-1+ T cells with a low proliferative and activation response (51). Here, we provide evidence that T cells present alterations in activation and exhaustion markers, with CD8+ cytotoxic T cells presenting low CD28+ and high PD-1 expression. These results reinforce previous data that observed an exhausted phenotype in lymphocytes after SARS-CoV-2 infection and provide a link between low cytokine production and disturbances in cellular immunity in post- COVID-19 patients. Furthermore, an interesting find by Loretelli and coworkers (2021) pointed that PD-1 blockade counteracts the post- COVID-19 immune dysfunction and restores ex vivo cytokine production and T- cell function, suggesting a potential pharmacological role of immune checkpoints on immunological abnormalities induced by SARS-CoV-2 infection (52). On the other hand, monocytes seem to return to values similar to controls during the recovery phase post- SARS-CoV-2 infection.

The role of physical activity on post-viral infection is poorly understood. Although a series of hypothesis or narrative reviews indicate that physical activity may contribute to counteract the immune abnormalities identified in long COVID-19, reports regarding chronic fatigue syndrome and long COVID-19 patients indicate that exercise may worsen immune-mediated symptoms (51). Our sample size did not allow us to divide and compare the groups according to the PAL recommended by the guidelines (>150 min of MVPA by week) or not (<150 min of MVPA by week); however, cautiously we were able to verify some important associations of MVPA with systemic markers. Mainly, the MVPA covariate adjusted IL-10, triglycerides, and leptin in the plasma of post- COVID-19 patients. MVPA also modulated the peripheral frequency of Treg cells and the expression of PD-1 in CD8+ T cells, although it abrogated the statistical effect in the analysis of TNF-α and IL-6 production by LPS- and PMA-stimulated PBMC of post- COVID-19 patients.

When we address the hormones and metabolic profile related with energy metabolism, we may observe that leptin, triglycerides, and leptin/VAT ratio parameters were higher in the post-COVID-19 group when compared with the control group, adjusted for MVPA. Although the body fat (%) data did not show statistical differences, the post-COVID-19 group had slightly higher body fat content (%) than the control group (31.91 ± 11.06 vs 28.61 ± 8.85 p adjusted= 0.086, respectively), which added to the changes found in parameters directly related to body fat (leptin, triglycerides, and leptin/VAT ratio). Therefore, we decided to categorize the groups in high and low body fat mass (**Supplementary Figure 2**). We found interesting data regarding a higher fat mass (%) with worsened leptin parameters in the post-COVID-19 group compared with control; however, this result is associated with the level of physical activity, since it disappeared after adjustment by MVPA.

### Gene expression and energy metabolism alterations

After PBMC stimulation with LPS, there was a decrease in Rev-erbα expression in the Post-COVID-19 group compared with the control. A circadian clock gene Rev-erbα negatively controlled several genes involved in innate immunity, such as *Il6*, *Cxcl11*, and *MCP-1* (53). Also, deletion of Rev-erbα enhanced TH17-mediated proinflammatory cytokine expression (54), suggesting that Rev-erbα acts concurrently between the circadian control and inflammatory pathways with anti-inflammatory properties. These results could favor the expression of proinflammatory genes and consequent increase of cytokine production; however, on the other hand, many genes related to the inflammatory and metabolic response in monocytes were not altered, such as NK-kβ, TLR-4, HIF-α, and beta adrenergic receptors in both groups. Future studies should explore other genes or pathways to clarify these findings.

Regarding energy metabolism, we observed a higher value in the LEAK state and lower OXPHOS (CI) in the post-COVID group compared with the control after adjustment by MPVA. Thus, mild to moderate COVID-19 appears to have a direct impact on the mitochondrial respiration of PBMCs. These data show that when we control the data for the physical activity level, the mitochondrial production of ATP by PBMC decreases. The decreases in mitochondrial respiration by carbohydrate- derived substrates suggest that cells are either relying on glycolytic metabolism or using alternative substrates for mitochondrial respiration (e.g., fatty acids). Ajaz et al. showed a mitochondrial dysfunction in PBMCs of patients hospitalized with COVID-19 and demonstrated an increased rate of glycolysis and utilization of glucose as the main substrate for energy production (55). A recent review has suggested that individuals trained exhibit a better mitochondrial function in immune cells, and this can be related with anti-inflammatory response (56). A possible cause would be that these cells increase the oxidation of glucose to compensate for inhibition of the alternative fuel pathway. Despite using a simplest methodology, the results of our study follow the same line of reasoning, which we could cautiously suggest that this dysfunction accompanies the post- COVID period, even with milder infections. Future studies should clarify how long these changes are maintained and possible negative repercussions.

The main strengths of our study are the comprehensive clinical and immunological outcomes analyzed, prior to the vaccination process. Additionally, the main limitation is that the proposed sample calculation was not achieved, explained by the moment the study was carried out, with the vaccination system in progress and constant change of public policies related to the management of the disease. We emphasize that it was not proposed to test causality and effect since the design of our study (cross-sectional) does not allow for this. Our intention was to explore possible associations of physical activity level with inflammatory and molecular responses in mild to moderate post-COVID-19.

## Conclusion

Young adults after mild-to-moderate SARS-CoV-2 infection appear to have lower physical activity levels, which can be associated with clinical and immunometabolic responses in a complex manner.

## Data availability statement

Data supporting of this study is available from (ZENODO) at (DOI: 10.5281/zenodo.8277005). Access to data is subject to approval by the authors, as the study (follow-up) is ongoing (DOI: 10.3390/ijerph182413249).

## Ethics statement

The study was approved by Research Ethics Committee of São Paulo State University (UNESP), Presidente Prudente, Brazil (approval number: 38701820.0.0000.5402). It was conducted in accordance with the local legislation and institutional requirements. The participants provided their written informed consent to participate in this study.

## Author contributions

Conceptualization: FL, TP, M-JC-E-S, AC, and BS. Methodology: AF, AM, AC, BS, VS, and OJ. Formal analysis: LM, AF, BS, CP, CF, TO-O, IS, JR, AT, GD, BM, and VS. Investigation: FL, TP, M-JC-E-S, AC, MS, RS, VL, JL, HI, KK, JR-N, and RP. Data curation: AF, CP, LM, GD, TO-O, and BS. Writing—original draft preparation: BS, FL, GD, and JR-N. Writing—review and editing: JR-N, MS, RS, TP, AC, KK, JL, HI, BS, and FL. Visualization: AF, FL, AM, TP, M-JC-E-S, AC, LM, CP, RP, and BS. Supervision: AF, FL, and BS. Project administration: FL and BS. Funding acquisition: FL. All authors have read and agreed to the published version of the manuscript.

## Supplementary material

Supplementary Figure 1 Rating of perceived exertion and functional parameters at rest, after 3, after 6 min, and recovery during 6MWT between control and Post-COVID-19 individuals. Data are presented as mean ± SD. SpO_2_: peripheral oxygen saturation. * p value set < 0.05 for bonferroni adjustment for multiple comparisons; # p value set <0.05 for bonferroni adjustment for multiple comparisons of performed using analysis of covariance (ANCOVA) with adjustment for MVPA. Upper symbols (*#): intragroup comparisons referring to the control group; lower symbols (*#): intragroup comparisons referring to the COVID-19 group.

Supplementary Figure 2 Categorization of groups by percentage of fat mass. Values expressed as mean ± SE. adj p: between group comparisons were performed using analysis of covariance (ANCOVA) with adjustment for MVPA. P value set < 0.05.

Supplementary Figure 3 Genes expression relative to B-tubulin in PBMC stimulated in absence or presence of of PMA [50 ng/mL] plus Ionomycin [1 µg/mL] between control and Post-COVID-19. Values expressed as mean ± SE of **(A)** TLR-4 expression, NF-κB expression, HIF-1α expression, α1 receptor expression, α2 receptor expression, BMAL1 expression, Rev-Erb-α expression (2^-ΔΔct). **B**) Respective genes percent changes for both stimulated condition (Control n=8; COVID-19 n=9). adj p: between group comparisons were performed using analysis of covariance (ANCOVA) with adjustment for MVPA. P value set < 0.05.

Supplementary Figure 4 Lymphocytes cultured in presence or absence of PMA [50 ng/mL] plus Ionomycin [1μg/mL] and monocytes cultured in presence or absence of LPS [10 ng/mL] between control and Post-COVID-19. Values expressed as mean ± SE of **(A)** IL-6 (pg/mL); TNF- α (pg/mL) (Control n=13; COVID-19 n=14) for control and LPS-stimulated condition in monocytes cultured and respective percent changes were compared for both stimulated condition; **(B)** IL-6 (pg/mL); TNF- α (pg/mL) (Control n=13; COVID-19 n=14) for control and PMA+ ionomycin-stimulated condition in lymphocytes cultured and respective percent changes were compared for both stimulated condition. adj p: between group comparisons were performed using analysis of covariance (ANCOVA) with adjustment for MVPA. P value set < 0.05.
