# Supplementary figures and images for: Mild to moderate post-COVID-19 alters markers of lymphocyte activation, exhaustion, and immunometabolic responses that can be partially associated by physical activity level— an observational sub-analysis fit- COVID study
Source: Front Immunol. 2023 Sep 11;14:1212745. doi: 10.3389/fimmu.2023.1212745 (PMC10518618; doi:10.3389/fimmu.2023.1212745)

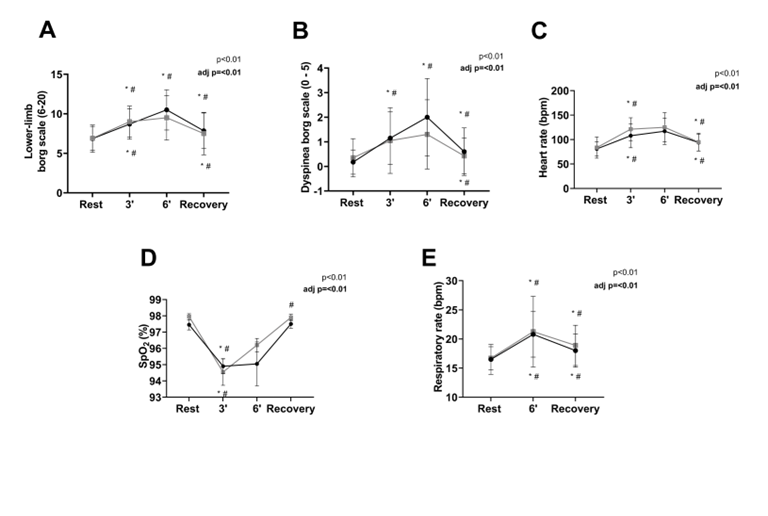

Supplement: Supplementary Figure 1 — Rating of perceived exertion and functional parameters at rest, after 3, after 6 min, and recovery during 6MWT between control and Post-COVID-19 individuals. Data are presented as mean ± SD. SpO2: peripheral oxygen saturation. * p value set < 0.05 for bonferroni adjustment for multiple comparisons; # p value set <0.05 for bonferroni adjustment for multiple comparisons of performed using analysis of covariance (ANCOVA) with adjustment for MVPA. Upper symbols (*#): intragroup comparisons referring to the control group; lower symbols (*#): intragroup comparisons referring to the COVID-19 group. [file Image_1.tif]

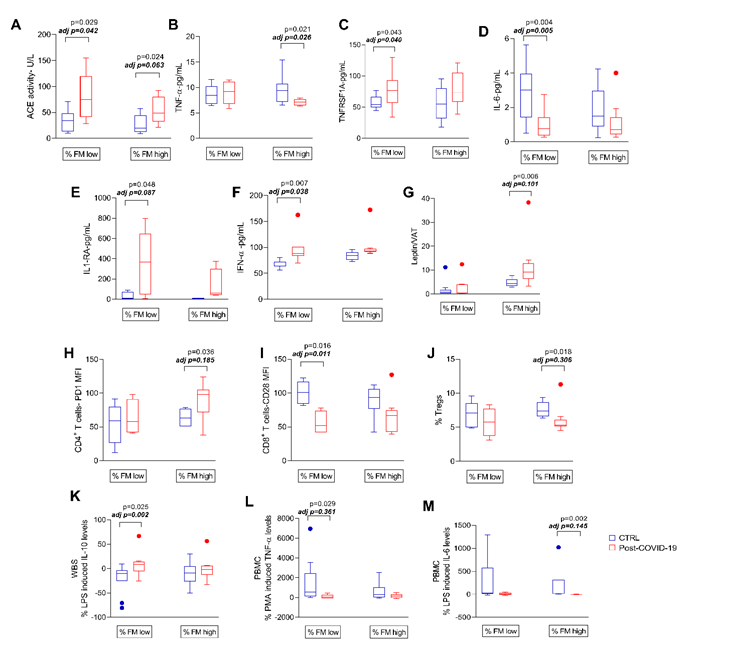

Supplement: Supplementary Figure 2 — Categorization of groups by percentage of fat mass. Values expressed as mean ± SE. adj p: between group comparisons were performed using analysis of covariance (ANCOVA) with adjustment for MVPA. P value set < 0.05. [file Image_2.tif]

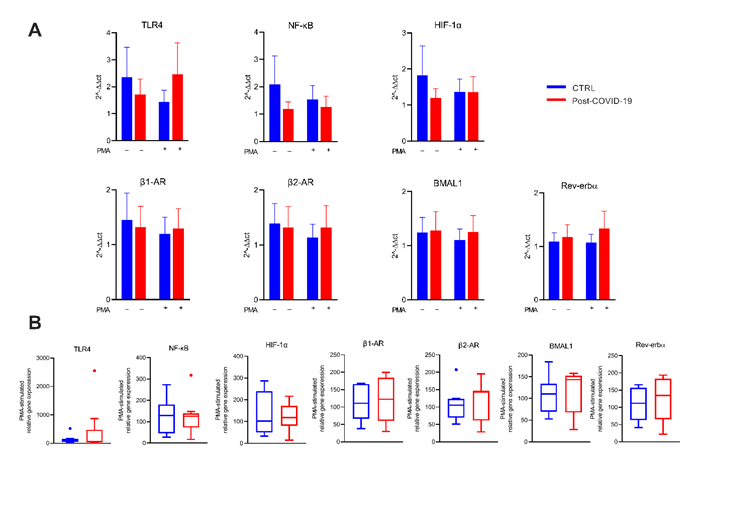

Supplement: Supplementary Figure 3 — Genes expression relative to B-tubulin in PBMC stimulated in absence or presence of of PMA [50 ng/mL] plus Ionomycin [1 µg/mL] between control and Post-COVID-19. Values expressed as mean ± SE of (A) TLR-4 expression, NF-κB expression, HIF-1α expression, α1 receptor expression, α2 receptor expression, BMAL1 expression, Rev-Erb-α expression (2^-ΔΔct). B) Respective genes percent changes for both stimulated condition (Control n=8; COVID-19 n=9). adj p: between group comparisons were performed using analysis of covariance (ANCOVA) with adjustment for MVPA. P value set < 0.05. [file Image_3.tif]

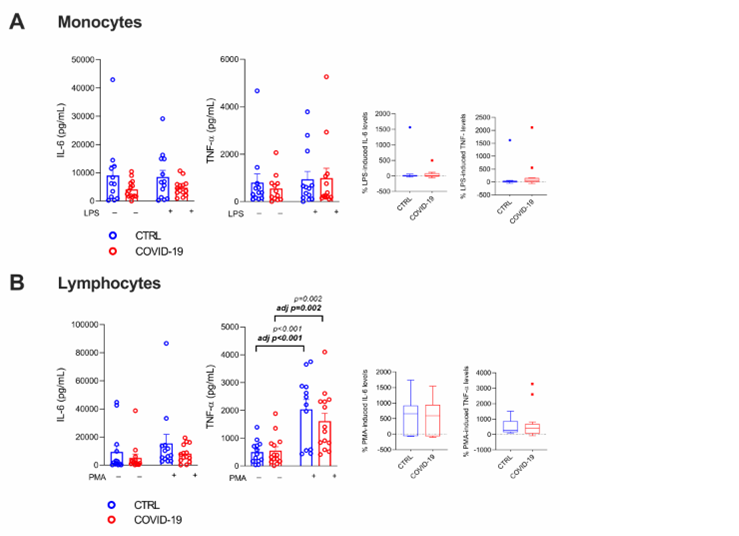

Supplement: Supplementary Figure 4 — Lymphocytes cultured in presence or absence of PMA [50 ng/mL] plus Ionomycin [1μg/mL] and monocytes cultured in presence or absence of LPS [10 ng/mL] between control and Post-COVID-19. Values expressed as mean ± SE of (A) IL-6 (pg/mL); TNF- α (pg/mL) (Control n=13; COVID-19 n=14) for control and LPS-stimulated condition in monocytes cultured and respective percent changes were compared for both stimulated condition; (B) IL-6 (pg/mL); TNF- α (pg/mL) (Control n=13; COVID-19 n=14) for control and PMA+ ionomycin-stimulated condition in lymphocytes cultured and respective percent changes were compared for both stimulated condition. adj p: between group comparisons were performed using analysis of covariance (ANCOVA) with adjustment for MVPA. P value set < 0.05. [file Image_4.tif]
