# Supplementary material for: Mild to moderate post-COVID-19 alters markers of lymphocyte activation, exhaustion, and immunometabolic responses that can be partially associated by physical activity level— an observational sub-analysis fit- COVID study
Source: Front Immunol. 2023 Sep 11;14:1212745. doi: 10.3389/fimmu.2023.1212745 (PMC10518618; doi:10.3389/fimmu.2023.1212745)
Supplement: Supplementary file 5 [file Table_1.docx]

**Supplementary Table 1.** Prime sequences of RT-PCR analysis.

| **Gene** | **Primer forward** | **Prime reverse** |
| --- | --- | --- |
| GAPDH | ACAACTTTGGTATCGTGGAAGG | GCCATCACGCCACAGTTTC |
| β-TUBULIN | TTGGGAGGTCATCAGCGATGAG | AGGCTCCAGATCCACCAGGATG |
| AMPK | GGCACGCATACCCTTGAT | TCTTCCTTCGTACACGCAAATAA |
| NF-κB | GAAGCACGAATGACAGAGGC | GCTTGGCGGATTAGCTCTTTT |
| TLR-4 | TTTATCCAGGTGTGAAATCCAG | AGATGCTAGATTTGTCTCCACAG |
| HIF-1α | AGTTCACCTGAGCCTAATAGTCC | TCCAAGTCTAAATCTGTGTCCTG |
| AR-β1 | TTCCTGCCCATCCTCATGCACT | GTAGAAGGAGACTACGGACGAG |
| AR-β2 | TACCAGAGCCTGCTGACCAAGA | AGTCACAGCAGGTCTCATTGGC |
| Bmal1-ARNTL | GCAGCTCCACTGACTACCAAG | TGTGAGCTTCCCTTGCATTT |
| REV-ERBα | CCCCAATGACAACAACACCT | CATAGGACATGCCAGCAGAAC |
