# Supplementary material for: Mild to moderate post-COVID-19 alters markers of lymphocyte activation, exhaustion, and immunometabolic responses that can be partially associated by physical activity level— an observational sub-analysis fit- COVID study
Source: Front Immunol. 2023 Sep 11;14:1212745. doi: 10.3389/fimmu.2023.1212745 (PMC10518618; doi:10.3389/fimmu.2023.1212745)
Supplement: Supplementary file 6 [file Table_2.docx]

**Supplementary Table 2.** Mental health and sleep quality of participants.

|  | **N** | **Control** | **N** | **Post-COVID-19** | ***p* value** | ***p a*djusted** |
| --- | --- | --- | --- | --- | --- | --- |
| ***Pittsburgh Sleep***  ***Quality Index*** | **20** |  | **17** |  |  |  |
| *Sleep duration* |  |  |  |  |  |  |
| ≥ 7 hours |  | 12 (60.00) |  | 12 (70.6) |  |  |
| < 7 hours |  | 8 (40.00) |  | 5 (29.4) |  |  |
| *Sleep efficiency* |  |  |  |  |  |  |
| *≥ 75%* |  | 14 (70.00) |  | 16 (94.1) |  |  |
| *< 75%* |  | 6 (30.00) |  | 1 (5.9) |  |  |
| *Use of sleep medications* |  |  |  |  |  |  |
| No |  | 20 (100) |  | 16(94.1) |  |  |
| Yes |  | 0 (0) |  | 1 (5.9) |  |  |
| *Sleep quality* |  |  |  |  |  |  |
| Poor |  | 13 (75.00) |  | 9 (52.9) |  |  |
| Good |  | 7 (35.00) |  | 8(47.10) |  |  |
| *Global score* |  | 6.6 ±3.53 |  | 5.41 ± 2.55 | 0.256 | 0.393 |
| ***Epworth Sleepiness Scale*** | **20** |  | **17** |  |  |  |
| Global score |  | 9.30 ± 3.54 |  | 8.88 ± 4.87 | 0.765 | 0.675 |
| ***Anxiety and Depression*** | **18** |  | **19** |  |  |  |
| ***HADS*** |  |  |  |  |  |  |
| Improbable |  | 3 (16.7) |  | 8 (42.10) |  |  |
| Possible |  | 9 (50.00) |  | 6 (31.6) |  |  |
| Probable |  | 6 (33.3) |  | 5 (26.3) |  |  |
| Total score |  | 10.50 ± 3.65 |  | 9.42± 4.50 | 0.430 | 0.504 |
| ***Beck Depression Inventory*** | **20** |  | **17** |  |  |  |
| Normal |  | 18 (90.00) |  | 15 (75.00) |  |  |
| Mild depression |  | 1 (5.00) |  | 1 (5.00) |  |  |
| Moderate depression |  | 1 (5.00) |  | 0 (0) |  |  |
| Severe depression |  | 0 (0) |  | 1 (5.00) |  |  |
| Total score |  | 9.50 (6.00 – 12.00) |  | 6 (4.5– 10.50) | 0.198 | 0.573 |
| ***Memory and attention*** |  |  |  |  |  |  |
| ***Digit Span test*** | **20** |  | **18** |  |  |  |
| Forward Digit Span |  | 8(7 – 9) |  | 6 (5 –8.25) | **0.035** | 0.055 |
| Reverse Digit Span |  | 5 (3.25 – 7) |  | 4 (3.75 – 5.25) | 0.228 | 0.357 |

**Note:** Data are presented as mean ± SD for data with normal distribution and median (IQR) for data with non-normal distribution HADS: hospital anxiety and depression scale. Bold values p < 0.05 compared with control. *P adjusted*: between group comparisons were performed using analysis of covariance (ANCOVA) with adjustment for MVPA.
